# Editorial: Imaging in glaucoma

**DOI:** 10.3389/fmed.2025.1744373

**Published:** 2026-01-12

**Authors:** Alberto Diniz-Filho, Andrew J. Tatham, Carolina P. B. Gracitelli, Lisandro M. Sakata

**Affiliations:** 1 Department of Ophthalmology and Otorhinolaryngology and Hospital São Geraldo – Hospital das Clínicas, Federal University of Minas Gerais, Belo Horizonte, Brazil; 2 Department of Ophthalmology and Princess Alexandra Eye Pavilion, University of Edinburgh, Edinburgh, United Kingdom; 3 Department of Ophthalmology and Visual Sciences and Hospital São Paulo, Federal University of São Paulo, São Paulo, Brazil; 4 Department of Ophthalmology and Hospital de Clínicas, Federal University of Paraná, Curitiba, Brazil

**Keywords:** angle-closure glaucoma, glaucoma, open-angle glaucoma, optical coherence tomography, optical imaging, visual field

Glaucoma is the leading cause of irreversible blindness worldwide, and it is estimated that by 2040, 111.8 million people between the ages of 40 and 80 will be affected by the disease (1). Imaging technologies offer the ability to objectively quantify glaucomatous damage, allowing for early diagnosis and monitoring of progression over time, thus enabling appropriate disease management. Since Huang et al. introduced Optical Coherence Tomography (OCT) in 1991 (2), using low-coherence interferometry to produce two-dimensional optical scattering images of the microstructures of internal ocular tissues, there has been a revolution in the structural assessment of retinal and optic nerve diseases through this technology. This Research Topic brings together four articles that cover different facets of imaging in glaucoma.

Li et al., using Swept-Source OCT (SS-OCT), demonstrated that the eyes of patients with primary open-angle glaucoma (POAG) and high myopia have the thinnest anterior scleral thickness, the shortest scleral spur, the thinnest trabecular meshwork, and the smallest Schlemm's canal compared to the eyes of patients with POAG and the eyes of patients with high myopia. Since the thinnest anterior scleral thickness might contribute to the shortest scleral spur and, consequently, to the thinnest trabecular meshwork and smallest Schlemm's canal in POAG eyes with high myopia, anterior scleral thickness may serve as a novel clinical indicator for the prediction and evaluation of POAG in high myopes. Furthermore, increased aqueous humor outflow resistance caused by the abnormal ocular anterior segment morphology in eyes of POAG patients with high myopia may also be a reason for the progression of visual field loss in this population.

In a retrospective case-control study conducted in the Hubei region, Wang et al. investigated the pathogenesis of primary angle-closure glaucoma (PACG) and its relationship to the anatomical structure of the anterior segment, obtaining biometric parameters through SS-OCT. They demonstrated that the eyes of patients with PACG have a smaller anterior segment space, narrower angles, a thicker lens, a thinner cornea, shorter axial length, a flatter cornea, and a more anteriorly positioned lens compared to the normal control group. The study highlights the significant discrepancies in anterior segment biometric parameters between patients with PACG and normal controls, reinforcing the notion that the pathogenesis of PACG is intrinsically linked to disparities in the anatomical configuration of the anterior segment. Since the eyes of PACG patients have a crowded anterior segment with a shallower anterior chamber depth and a narrowed angle, shorter axial length, and a thicker and more anterior lens, it is reasonable to emphasize the importance of the assessment of anterior segment biometric parameters using SS-OCT to stratify the risk of developing angle closure in at-risk eyes, enabling early intervention.

Although perimetry is a functional test and not an imaging modality in glaucoma itself, the automated and precise localization of predicted structural and functional information is now possible with new technologies, serving as a crucial initial step toward a more integrated diagnosis and monitoring of glaucoma progression in terms of structure and function. That being said, Al-Nosairy et al. explored a novel kinetic method for visual field screening called rapid campimetry. It was designed to screen the central 10 degrees of the visual field, an important region in glaucoma. Rapid campimetry was found to be robust even under suboptimal testing conditions and demonstrated good to excellent reliability between testing visits, which highlights its promise as a screening test and potential upon proper future optimization.

The potential role of the microvasculature and blood flow in the pathophysiology of glaucoma has been extensively debated and investigated. In this context, OCT Angiography (OCTA) has emerged as an invaluable non-invasive imaging modality used to characterize the vasculature in retinal layers, supplying detailed data on the microvasculature of the optic nerve head and retina (3). Previous studies have demonstrated that OCTA vessel density is lower in glaucoma patients and is highly correlated with visual field indices (4–6). Shen et al. presented a comprehensive overview of OCTA applications in glaucoma, showing its current status and future directions. They also explored the role of OCTA in detecting, monitoring, and predicting glaucoma progression. The clinical translation of OCTA opens up a promising avenue for the early diagnosis, the monitoring of progression, and the assessment of treatment outcomes in glaucoma.

Advances in this field can help us continue to make significant progress in reducing the global impact of irreversible vision loss from glaucoma. For example, integrating artificial intelligence (AI) strategies with glaucoma imaging into clinical practice will enable the provision of high-quality care to a population that continues to increase in number and age (7). Considering the shortcomings related to the lack of objective criteria for glaucoma diagnosis, standardized guidelines aimed at clearly classifying images for glaucoma detection and monitoring can pave the way for the development of reliable AI models (8). The future incorporation of AI into healthcare models can help overcome current limitations in access to and timely treatment of glaucoma patients worldwide. Each of the above studies demonstrates the potential utility of imaging in glaucoma, and we hope that the articles in this Research Topic will contribute to the knowledge in this field.
